# GPRC6a is not Required for the Effects of a High-Protein Diet on Body Weight in Mice

**DOI:** 10.1002/oby.21083

**Published:** 2015-05-09

**Authors:** James S Kinsey-Jones, Amin Alamshah, Anne K McGavigan, Eleanor Spreckley, Katherine Banks, Nicholas Cereceda Monteoliva, Mariana Norton, Gavin A Bewick, Kevin G Murphy

**Affiliations:** 1 Section of Investigative Medicine, Department of Medicine, Imperial College London, Hammersmith HospitalLondon, UK; 2 Division of Diabetes and Nutritional Sciences, King's College LondonGuy's Campus, London, UK

## Abstract

**Objective:**

The G-protein coupled receptor family C group 6 member A (GPRC6A) is activated by proteinogenic amino acids and may sense amino acids in the gastrointestinal tract and the brain. The study investigated whether GPRC6A was necessary for the effects of low- and high-protein diets on body weight and food intake in mice.

**Methods:**

The role of GPRC6A in mediating the effects of a low-protein diet on body weight was investigated in GPRC6a knockout (GPRC6a-KO) and wild-type (WT) mice fed a control diet (18% protein) or a low-protein diet (6% protein) for 9 days. The role of GPRC6A in mediating the effects of a high-protein diet on body weight was investigated in GPRC6a-KO and WT mice fed a control diet (18% protein) or a high-protein diet (50% protein) for 5 weeks.

**Results:**

A high-protein diet reduced body weight gain and food intake compared with a control diet in both WT and GPRC6a-KO mice. A low-protein diet decreased body weight gain in GPRC6a-KO mice.

**Conclusions:**

GPRC6A was not necessary for the effects of a low- or high-protein diet on body weight and likely does not play a role in protein-induced satiety.

## Introduction

Protein has a powerful satiating effect that is greater than that of carbohydrate or fat (1). High-protein diets reduce food intake, facilitate weight loss, and improve body composition in both humans and animal models (2,3), but they are difficult to adhere to. Rodents have been shown to avoid low-protein diets, resulting in reduced body weight when *ad libitum* fed such a diet (4). In contrast, feeding rodents diets moderately low in protein can cause initial periods of reduced food intake, followed by a subsequent increase in food intake and body fat which is thought to reflect a drive to increase protein intake (5,6). Diets containing disproportionate amounts of specific amino acids can also result in changes in food intake (7). However, although several possibilities have been proposed (8), the precise mechanisms by which protein intake is sensed in order to modulate appetite remain to be elucidated (9). Exploiting the systems by which protein suppresses appetite may be a viable approach to the treatment of obesity (2).

The digestion of proteins results in the production of small peptides and amino acids in the lumen of the gastrointestinal tract. Recent evidence suggests that l-amino acids may be sensed by a group of promiscuous G-protein coupled receptors which include the T1R1/T1R3 heterodimer, the calcium sensing receptor (CaSR), and the G-protein coupled receptor family C group 6 member A (GPRC6A). Both human and mouse GPRC6A orthologs are activated by basic l-amino acids, including arginine, lysine, ornithine, and several aliphatic amino acids (10). Arginine and lysine have been demonstrated to reduce appetite in rodents, though the mechanisms by which they mediate these effects are unclear (11). GPRC6A is expressed in a wide range of tissues (12), suggesting it may serve a diverse range of physiological functions, and there is interest in GPRC6A as a potential novel target in therapeutic areas such as metabolism, obesity, and endocrine function (13).

GPRC6a knockout (GPRC6a-KO) mouse models have previously been used to investigate the physiological roles of GPRC6A. Pi et al. generated the first global GPRC6a-KO mice by targeted deletion of exon II (GPRC6a^exon 2(−/−)^) (14). Exon II encodes for a minor part of the venus fly trap domain of the receptor which contains an orthosteric binding site for endogenous ligands. This mouse is reported to have multiple physiological abnormalities, including disrupted bone metabolism, feminization of the male mice, adiposity, and glucose intolerance. Wellendorph et al. subsequently described another GPRC6a-KO mouse, in which exon VI, containing the entire 7 transmembrane and C-terminal region of the receptor, was disrupted (GPRC6a^exon 6(−/−)^) (15). In contrast to the GPRC6a^exon 2(−/−)^ model, the GPRC6a^exon 6(−/−)^ model has been reported to have a normal bone phenotype and normal glucose tolerance (15,16).

GPRC6a^exon 6(−/−)^ mice with *ad libitum* access to standard chow diet have normal food intake and body weight progression (15), but GPRC6a^exon 2(−/−)^ mice on the same diet have increased adiposity (14). However, a recent study demonstrated that GPRC6a^exon 6(−/−)^ mice show a similar phenotype when placed on a high-fat diet, becoming hyperphagic and exhibiting increased body weight and impaired glucose homeostasis (17). Therefore, despite the differences between the knockout models, evidence suggests a role for GPRC6A in energy homeostasis and metabolism.

GPRC6A expression has been reported in the alimentary system, specifically within gastrin expressing cells of the stomach (18), and in pancreatic tissue, and it has also been detected in the mouse TC-6 pancreatic *β*-cell line (19). It has been suggested that GPRC6A mediates the effects of the amino acid l-arginine on insulin release from mouse pancreatic islet (20), though this has not been found by all groups (16). In addition, the bone-derived hormone osteocalcin has been reported to enhance GPRC6A activity (21), though in a recent study another group were not able to demonstrate osteocalcin agonism at GPRC6A (22). Osteocalcin promotes beta cell proliferation and can stimulate insulin release directly and indirectly via the release of glucagon-like peptide-1 (GLP-1) from enteroendocrine cells, and these effects are thought to be mediated by GPRC6A, as demonstrated using an exon II pancreas-specific GPRC6a-KO mouse model (23). GLP-1 expressing L cells in the intestine sense nutrients in order to regulate glucose and energy homeostasis (24). This data is thus in accord with suggestions that GPRC6A acts as a nutrient sensor in the digestive system (25), though the relative expression of GPRC6A in different regions of the gut has not been reported. It has been proposed that GPRC6A may sense amino acids in order to detect protein ingestion and modulate food intake accordingly and thus may be a useful target for pharmacological agents to treat obesity (13,26). However, the physiological role of GPRC6A in mediating the response to altered protein intake is unknown. We investigated the acute and chronic effects of altered dietary protein concentrations on food intake and body weight in wild-type (WT) and GPRC6a*-*KO mice to assess the putative role played by GPRC6A in modulating protein-induced changes in energy homeostasis.

## Methods

### Animals

The GPRC6a-KO model used in our studies was obtained from the Knockout Mouse Project (KOMP) (27). The C57BL/6NTac inbred mouse was used as the genetic background for developing the GPRC6a-KO congenic strain (Taconic, USA). The deleted region consisted of 16,596 base pairs, which completely covers the GPRC6a locus. GPRC6a-targeted heterozygous C57B1/6N mice were bred to produce homozygous GPRC6a-KO animals and WT littermate controls. All experiments were conducted in 6-8 week old male mice individually housed on a 12  h light: 12  h darkness cycle under controlled temperature and humidity. We found no difference in basal glucose levels or in the response to an intraperitoneal glucose tolerance test (IPGTT) between GPRC6a-KO animals and WT controls (Supporting Information Figure S1). All animals were weight-matched prior to the start of the chronic studies. Animal procedures were approved under the UK Home Office Animals (Scientific Procedures) Act 1986.

### GPRC6a expression in the gastrointestinal tract

A cohort of WT and GPRC6a-KO mice fed RM1 standard chow diet (RM1 diet; Special Diet Services, Witham, Essex, UK) were fasted overnight to avoid any effects of acute food intake on gene expression. Mice were then decapitated and stomach, duodenum, jejunum, ileum, and colon rapidly removed, snap frozen, and stored at −80°C for RNA extraction and qPCR. Total RNA was extracted from gastrointestinal tissues using TRI reagent (Sigma-Aldrich, Poole, UK) in accordance with the manufacturer's instructions. Reverse transcription was carried out using the High Capacity cDNA Reverse Transcription Kit (Life Technologies, Paisley, UK) according to the manufacturer's instructions. Real-time quantitative PCR analysis was performed using TaqMan Gene Expression Assays and TaqMan Universal PCR Master Mix (Life Technologies, Paisley, UK) using the ABI Prism 7900 Sequence Detection System according to the protocols provided by the manufacturer (Life Technologies, Paisley, UK). The relative mRNA transcript levels were calculated according to the 2^–ΔCT^ method, with ΔCT being the difference in cycle threshold values between the GPRC6a mRNA (Mm00467618_m1), T1R1 mRNA (Mm00473433_m1), T1R3 mRNA (Mm00473459_g1) or CaSR mRNA (mCG130161), and the hypoxanthine phosphoribosyltransferase 1 (HPRT1) mRNA (m00446968_m1) internal control. GPRC6a expression was also measured using the same methodology in tissues from WT mice from the chronic high protein intake study described below. For these studies, expression analysis was carried out at the end of the 5-week-high-protein diet feeding. The animals were fasted overnight prior to the cull and tissue collected as described above.

### Acute and chronic effects of high protein intake in GPRC6a-KO mice

The effects of an acute protein load on food intake were compared in WT and GPRC6a-KO mice in a randomised paired cross over study. Animals were orally gavaged with water or cooked chicken breast (Supporting Information Table S1B) homogenised in water (2 g/kg) during the early light phase following an overnight 16 h fast. The chicken breast was pressure cooked, the fat removed by centrifugation, and the chicken then homogenised in water according to a previously published method (26). Food intake was measured at 1, 2, 4, 8, and 24 h postadministration.

To examine the role of GPRC6A in sensing chronically elevated protein intake, WT control and GPRC6a-KO mice were given *ad libitum* access to an 18% protein control diet or a 50% high-protein diet (HP) (Supporting Information Table S1A) (Harlan Teklad, Oxfordshire, UK). Diets were isocaloric and matched for fat content (Supporting Information Table S1A). Body weight and food weight were measured at least three times a week for 5 weeks. Animals were then fasted overnight, culled and tissues collected from different regions of the gastrointestinal tract for RNA extraction.

### Effects of a low-protein diet in GPRC6a-KO mice

To investigate the potential role of GPRC6A in mediating the response to low protein intake, WT control, and GPRC6a-KO mice were given *ad libitum* access to an 18% protein control diet (Supporting Information Table S1A) or a 6% low protein diet (LP) (Supporting Information Table S1A) (Harlan Teklad, Oxfordshire, UK). Diets were isocaloric and matched for fat content (Supporting Information Table S1A). Body weight and food intake were measured on days 1-5 and 8-9.

### Statistical analysis

All values are mean ± SEM unless otherwise stated. Differences in cumulative food intake and body weight data through time were compared across experimental groups using generalized estimating equation curve analysis (Stata 9.1; Statacorp, College Station, TX). All other comparisons were made using either one way ANOVA followed by Tukey's test *post hoc* comparisons or multiple *t* test (Prism 6.03, GraphPad Software Inc). *P* < 0.05 was considered significant.

## Results

### GPRC6a expression in the gastrointestinal tract

**Figure 1:**
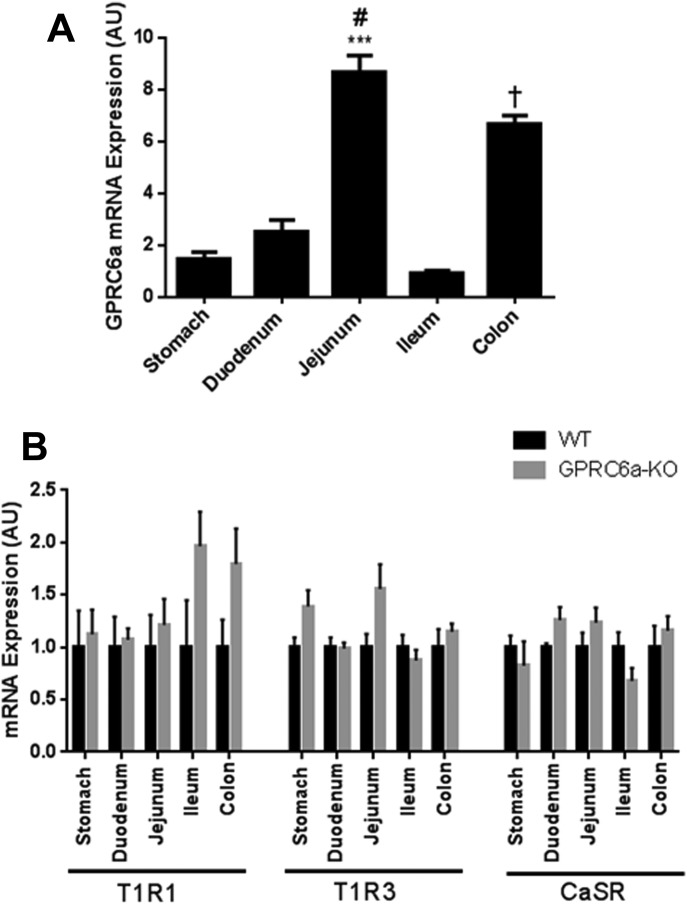
(A) Relative expression of GPRC6a mRNA in different regions of the gastrointestinal tract of overnight fasted male WT mice fed a control diet. Data is presented as mean ± SEM. ***P < 0.001 vs. stomach, duodenumn and ileum. ^#^*P* < 0.01 vs. colon. ^†^*P* < 0.001 vs. stomach, duodenum, and ileum. *n* = 7. (B) Relative expression of T1R1, T1R3, and CaSR mRNA in different regions of the gastrointestinal tract in male WT and GPRC6a-KO mice fed a control diet. The expression levels are relative to WT mice. Data is presented as mean ± SEM. *n* = 4–6 per group.

We examined the expression of GPRC6a in different levels of the gastrointestinal tract in mice. GPRC6a expression was detected in all regions of the gastrointestinal tract investigated, with the highest expression observed in the jejunum and colon (Figure 1A).

In addition to GPRC6A, CaSR and T1R1-T1R3 receptor complexes have been suggested to mediate amino acid sensing in the gut (28). It is therefore possible that the expression of CaSR and/or T1R1-T1R3 may be upregulated to compensate for the absence of GPRC6A expression. In order to investigate whether deletion of GPRC6A alters the expression of these receptors, we measured their expression in both WT and GPRC6a-KO mice. There were no significant differences in the expression of T1R1, T1R3, or CaSR in the regions of the gastrointestinal tract examined between WT or GPRC6a-KO mice, suggesting that expression of these receptors is not altered in the gut to compensate for the loss of GPRC6A (Figure 1B).

### Acute and chronic effects of high protein intake in GPRC6a-KO mice

**Figure 2:**
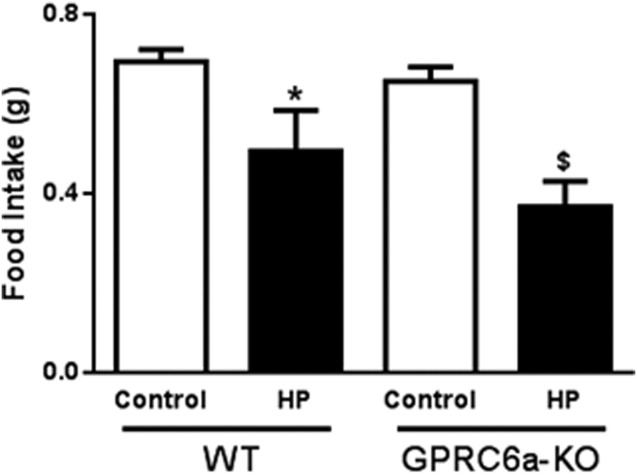
The effect of oral administration of a protein load (HP) on acute 0–1 h food intake in the early light phase following an overnight fast in GPRC6a-KO mice. Data is presented as mean ± SEM. *P < 0.05, HP vs. control in WT mice. ^$^*P* < 0.05, HP vs. control in GPRC6a-KO mice. *n* = 4–5 per group.

To investigate the role of GPRC6A in mediating the effects of high protein intake, WT and GPRC6a-KO mice were orally administered with a vehicle control or a high protein load. Acute oral administration of a protein load significantly reduced food intake in both WT (0.694 ± 0.027 g control vs. 0.496 ± 0.089 g high protein load) and GPRC6a-KO 0.650 ± 0.032 g control vs. 0.370g 0.0576 high protein load) mice in the 0-1 h period following administration (*P* < 0.05) (Figure 2). This was an acute affect, with no significant suppression of food intake observed in WTs or GPRC6a-KO animals in subsequent time intervals monitored up to 24 h following administration (data not shown).

**Figure 3:**
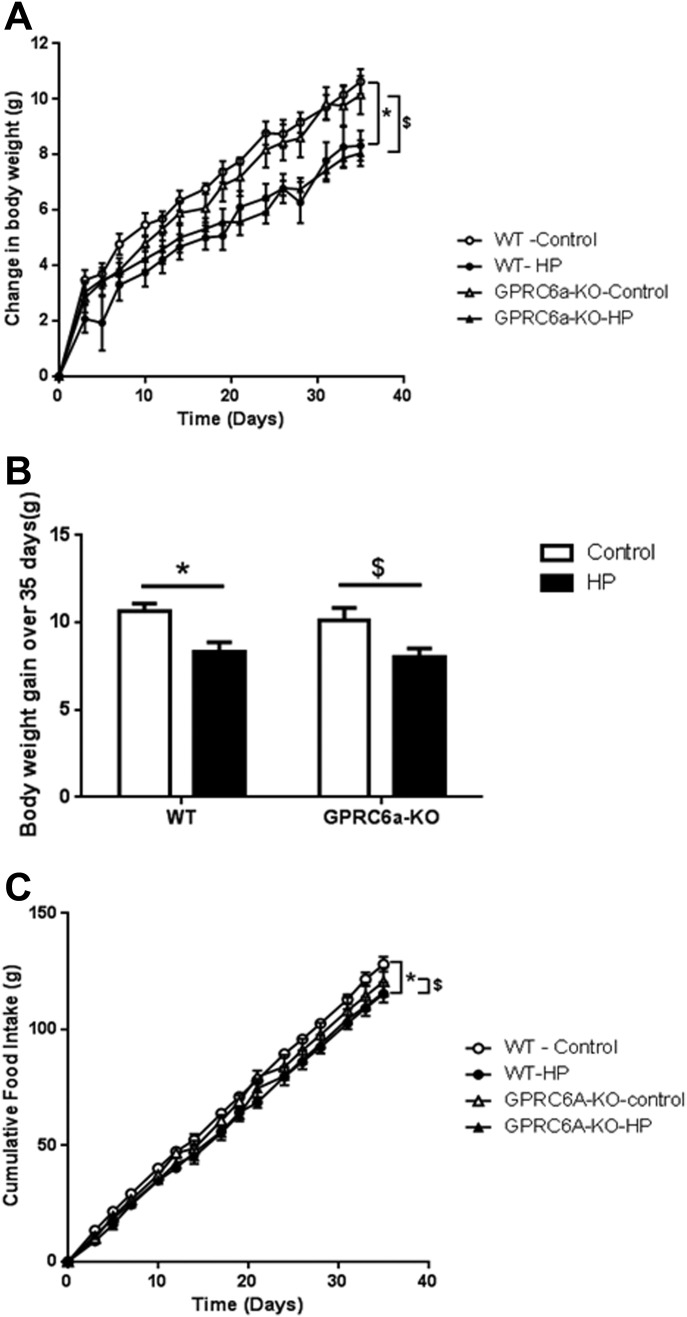
The effect of a high-protein (HP) diet on (A, B) body weight gain and (C) cumulative food take in WT and GPRC6a-KO mice. Data is presented as mean ± SEM. (A) and (C): *P < 0.05, HP diet vs. control diet in WT mice. ^$^*P* < 0.05, HP diet vs. control diet in GPRC6A mice determined using generalized estimating equation curve analysis for the entire 5 weeks. (B): *P < 0.05, HP diet vs. control diet in WT mice. ^$^*P* < 0.05, HP diet vs. control diet in GPRC6A mice determined using multiple *t* test. *n* = 6–7 per group.

To determine whether GPRC6A is necessary for the chronic suppression of food intake and body weight driven by high protein (29), we placed WT and GPRC6a-KO mice on either a high-protein or a control diet for 5 weeks. There was no significant difference between the absolute body weights of animals between diet or genotype groups (day 0: 21.68 ± 0.90 g WT control vs. 22.41 ± 0.87 g WT-HP, 21.05 ± 1.18 g GPRC6a-KO-control vs. 23.30 ± 1.37 g GPRC6a-KO-HP, *P* > 0.05; day 35: 31.96 ± 0.98 g WT control vs. 30.74 ± 0.95 g WT-HP, 30.91 ± 1.50 g GPRC6a-KO-control vs. 31.35 ± 1.35 g GPRC6a-KO-HP, *P* > 0.05). However, *ad libitum* access to a high-protein diet significantly reduced body weight gain (Figure 3A,B) and food intake (Figure 3C) in both GPRC6a-KO mice and age-matched WT controls (*P* < 0.05).

**Figure 4:**
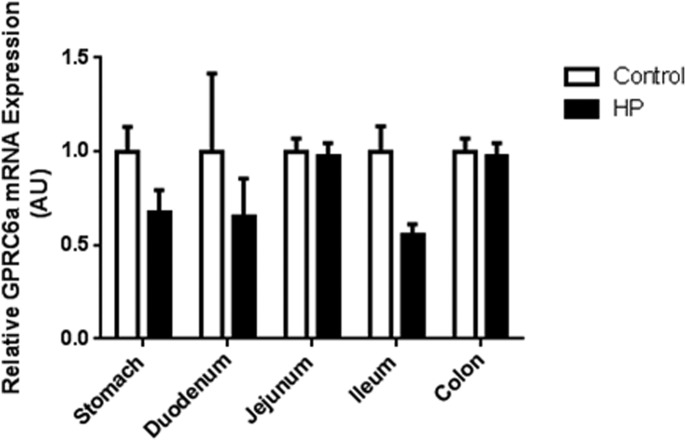
Relative expression of GPRC6a mRNA in different regions of the gastrointestinal tract of mice fed either a control or high-protein (HP) diet for 5 weeks, and then fasted overnight. GPRC6a mRNA expression is shown as expression relative to control diet. Data is presented as mean ± SEM. *n* = 7.

Expression levels of the receptors involved in the physiological response to ingested protein might be expected to adapt to a chronic high-protein diet. To investigate whether a high-protein-diet modulates the expression of GPRC6A in the gastrointestinal tract, we measured GPRC6a mRNA expression in different regions of the gastrointestinal tract of WT mice exposed to a control or a high-protein diet for 5 weeks. There was no significant difference in GPRC6a expression in WT mice exposed to a high-protein diet compared to those fed a control diet in any of the regions of the gastrointestinal tract examined (Figure 4).

### Effects of a low-protein diet in GPRC6a-KO mice

**Figure 5:**
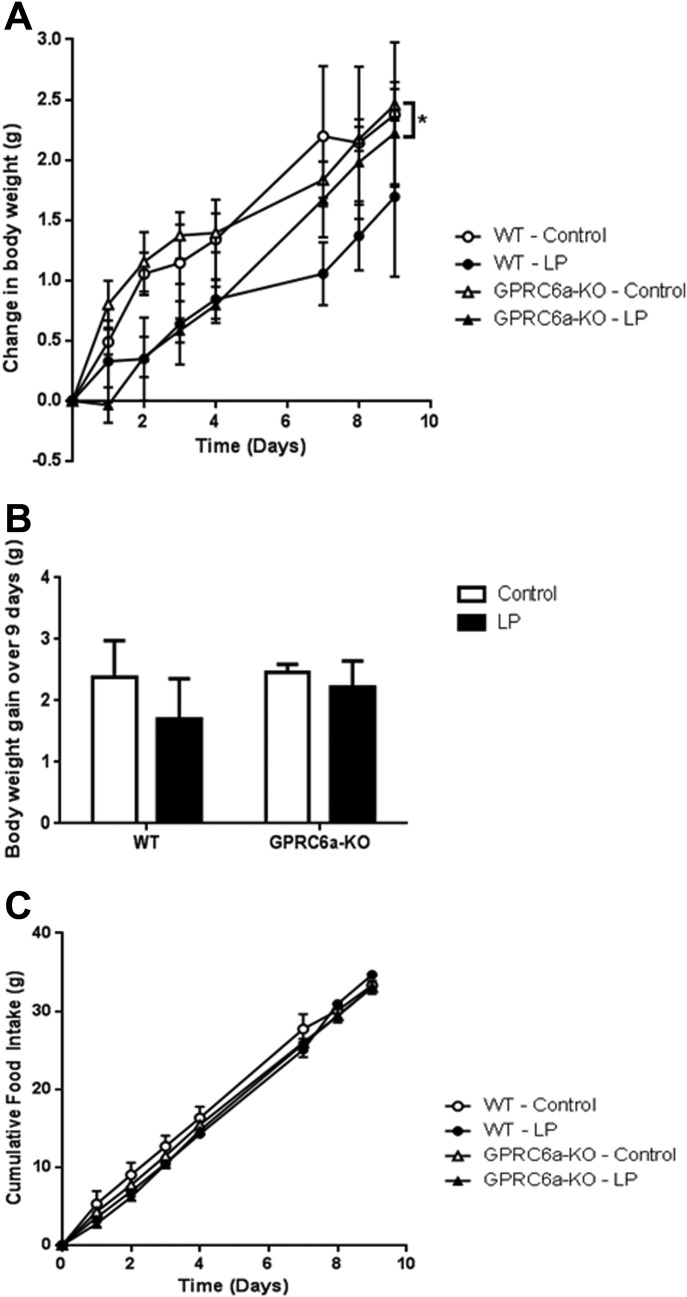
The effect of a low-protein diet (LP) on (A, B) body weight gain and (C) cumulative food intake in male WT and GPRC6a-KO mice. Data is presented as mean ± SEM. *P < 0.05 vs. LP diet group determined using generalized estimating equation curve analysis for the entire 9 days. *n* = 7–8 per group.

In order to investigate the potential role of GPRC6A in mediating the effects of a low-protein diet in mice, we placed both WT and GPRC6a-KO mice on either low-protein or control diets for 9 days. It has been shown that mice initially lose weight when placed on a low-protein diet due to reduced food intake (30), though there is often a subsequent increase in food intake (5,6). There was no significant difference between the absolute body weights of animals between diet or genotype groups (day 0: 21.65 ± 1.98 g WT control vs. 22.66 ± 1.32 g WT-LP, 22.32 ± 0.98 g GPRC6a-KO-control vs. 22.27 ± 1.07 g GPRC6a-KO-LP, *P* > 0.05; day 9: 24.02 ± 1.44 g WT-control vs. 24.35 ± 0.70 g WT-LP, 24.78 ± 1.00 g GPRC6a-KO-control vs. 24.50 ± 0.81 g GPRC6a-KO-LP, *P* > 0.05). However, GPRC6a-KO mice fed a low-protein diet displayed reduced body weight gain compared to GPRC6a-KO mice on a control diet. Although WT animals fed a low-protein diet had lower average body weight gain, this effect was not significant (Figure 5A,B). Both WT and GPRC6a-KO mice fed a low-protein diet had a lower average food intake compared to those fed an 18% protein diet, but these effects did not achieve statistical significance (Figure 5C). These observations suggest that GPRC6A is not necessary for the effects of a low-protein diet on body weight and food intake.

## Discussion

GPRC6A can act as a nutrient sensing receptor detecting L-amino acids and has been suggested to regulate energy homeostasis in response to protein intake. As such it has been proposed as a putative target for antiobesity agents (13,31). Our data demonstrates that while GPRC6A is expressed throughout the murine gastrointestinal tract, it is not necessary for the effects of low- or high-protein diets on energy homeostasis in mice. GPRC6a ablation had no impact on the effects of low- or high-protein diets in mice. We observed reductions in food intake and body weight in both WT and GPRC6a-knockout mice when animals were placed on low- or high-protein diets.

Rodents typically exhibit a reduction in body weight and food intake when placed on a high-protein diet (29). In rats, consumption of a high-protein diet for several days reduces cumulative food intake, weight gain, and adiposity compared to a normal-protein diet control (32). Two different GPRC6a knockout models have been generated to investigate the molecular signaling and physiological role of the receptor (13). These knockout models were generated by two different molecular approaches which consisted of partial deletion of GPRC6a exonic regions coding either the receptor seven transmembrane region (15) or part of the ligand binding site (14). Although Pi et al. reported differences in basal blood glucose levels and response to an IPGTT in their GPRC6a^exon 2(−/−)^ model (14), Smajilovic et al. observed no differences in basal blood glucose levels or in the response to an oral GTT in their GPRC6a^exon 6(−/−)^model (16). We found no differences in basal glucose levels or in the response to an IPGTT between WT and our GPRC6a-KO model, in accord with the phenotype of the GPRC6a^exon 6(−/−)^ model. While discrepancies in the phenotypes of these models have been reported, data from both suggests a possible role for GPRC6A in energy homeostasis (14,17). The GPRC6A knockout model used in present studies has the entire GPRC6a gene deleted. These studies therefore demonstrate that GPRC6A is not necessary for the effects of low- or high-protein diets on body weight.

The length of exposure to diets with altered protein content can influence the effects observed. We subjected mice to 9 and 35 days of low- or high-protein diets, respectively. However, it is possible that a longer exposure might have revealed differences between the cohorts. For example, a recent study investigating mice lacking GPRC6a found they required exposure to a high-fat diet for over 4 months to demonstrate a significant difference in body weight compared to WT controls, and only detected differences in percentage body fat after 25 weeks (17).

Mice generally lose weight when initially exposed to low-protein diets (30). In our WT mice, this effect did not achieve statistical significance. Previous rodent studies have also demonstrated an increased food intake and body fat associated with moderately low levels of dietary protein (5,6). The ratio of macronutrients and amino acids in the diet can also modulate energy homeostasis. For example, a recent study suggests that increased circulating levels of hepatic fibroblast growth factor 21 (FGF21) function as a specific endocrine signal of protein restriction in rodents, and mediate behavioral and metabolic responses to protein restriction (33). The altered amino acid content in our experimental diet may therefore have had subtle effects on energy homeostasis that were not detected in our studies, and which may have differed between GPRC6a knockout and WT mice.

There may be multiple overlapping systems mediating the effects of dietary amino acids and proteins on food intake (2). The deletion of a single receptor, such as GPRC6A, may not therefore be sufficient to prevent the effects of altered dietary protein content on food intake. The T1R1/T1R3 receptor heterodimer and the CaSR have also been suggested to be involved in amino acid sensing in the gut. Recent studies suggest T1R1/T1R3 may act as a sensor of amino acid availability that regulates the activity of the mammalian target of rapamycin (34). Specific l-amino acids can stimulate anorectic gut hormone release from isolated I-cells and rat small intestine through a CaSR-dependent mechanism (35,36). Furthermore, a recent study has demonstrated that GPRC6A is involved in l-amino acid-induced GLP-1 secretion from the GLUTag enteroendocrine cell line (31). Deletion of GPRC6a did not result in compensatory changes in expression of the other two promiscuous l-amino acid receptors in the gastrointestinal tract, though it is possible that the activity of these receptors may be altered by, for example, altering their localization to the cell membrane. In addition, exposure to a high-protein diet for 5 weeks seemed to have no effect on the expression of GPRC6a in gastrointestinal tract (Figure 4). However, it is possible that there were changes in GPRC6A protein concentrations, even though we detected no significant change in GPRC6a mRNA expression. Further studies investigating this possibility will require the use of antibodies with demonstrated specificity for GPRC6A.

Alternatively, other protein sensing mechanisms may compensate for the absence of GPRC6A. GPR93, a G-protein coupled receptor of the family A, is highly expressed on cholecystokinin (CCK) releasing I cells in small intestine, and has been implicated in the sensing of protein hydrolysate in the gastrointestinal lumen (37). Peptone stimulates the release of CCK from STC-1 cells via a GPR93 dependent mechanism (38). Mu-opioid receptors (MOR) have recently been demonstrated to play a role in protein sensing. MORs in neurons in the portal vein walls sense peptides in the blood which are released into the portal circulation following ingestion of a high-protein meal. These peptides antagonise MORs, regulating a gut-brain neural circuit that ultimately controls satiety and food intake (39). Amino acid transporter systems may also be implicated in protein sensing. Evidence suggests that in addition to their transport role, amino acid transporters have receptor-like properties, regulating nutrient induced signalling by sensing cellular amino acid sufficiency. For example, the sodium-coupled neutral amino acid transporter 2 (SNAT2) has shown to have transceptor activity and been implicated in glutamine-stimulated GLP-1 release from intestinal L-cells (40). We used cooked chicken breast as a model of high-protein food in our acute high-protein study (Figure 2) and observed an acute reduction in food intake in mice orally gavaged with chicken breast compared to water control. Although different protein loads may have different chronic effects on food intake and body weight, acute satiating effects can be difficult to detect in mice, and therefore require large cohorts of animals to investigate. Although water does not control for the differences in energy intake, it was used in our acute study to control for the initial changes in stomach distention caused by administration of the homogenised chicken breast.

The expression of GPRC6A in the gut, its activation by L-amino acids, and its putative ability to mediate hormone release has suggested that GPRC6A is a promising candidate for mediating the anorectic effects of altered dietary protein. However, our studies suggest that GPRC6A does not play a critical role in protein-mediated changes in appetite and body weight and thus may not be involved in protein sensing in the gut.
