# Supplementary material for: GPRC6a is not Required for the Effects of a High-Protein Diet on Body Weight in Mice
Source: Obesity (Silver Spring). 2015 May 9;23(6):1194–200. doi: 10.1002/oby.21083 (PMC4692088; doi:10.1002/oby.21083)
Supplement: Supplementary file 1 — Supplementary Information [file oby0023-1194-sd1.docx]

**Supplementary Material for ‘GPRC6A is not required for the effects of a high protein diet on body weight in mice’.**

**Authors:** James S Kinsey-Jones^1^, Amin Alamshah^1^, Anne K. McGavigan^1^, Eleanor Spreckley ^1^, Katherine Banks^1^, Nicholas Cereceda Monteoliva^1^, Mariana Norton^1^, Gavin A. Bewick^1, 2^, and Kevin G. Murphy^1^

^1^Section of Investigative Medicine, Imperial College London, London, UK.

^2^Division of Diabetes & Nutritional Sciences, King's College London, Guy's campus, London, UK.

**Corresponding author:** Dr KG Murphy, Section of Investigative Medicine, Department of Medicine, Imperial College London, Commonwealth Building, Hammersmith Hospital, Du Cane Road, London, W12 0NN, UK. E-mail: [k.g.murphy@imperial.ac.uk](mailto:k.g.murphy@imperial.ac.uk)

**Supplementary methods**

**Glucose tolerance test:**

Six to eight week old individually housed male mice were fasted for 16 hours overnight before an intraperitoneal injection of 20% glucose (2 g/kg). Samples for blood glucose were taken from the tail vein immediately before glucose injection (t = 0) and at 15, 30, 60 and 120 minutes following administration and were measured using a 65 glucometer (CONTOUR meter and test strips, Bayer, Berkshire, UK).

**Supplementary Figure 1. Intraperitoneal glucose tolerance test.**

**Supplementary Figure 1**. Glucose tolerance test (GTT) in male WT and GPRC6a-KO mice. Mice were fasted overnight and received an intraperitoneal injection of 20% glucose solution (2g/Kg body weight). Data is presented as mean ± SEM. n = 4 per group.

**Supplementary Table 1. Diets composition and nutrient information.**

1. **Protein diets**

| **Diets** | **TD.90016 6% Protein diet** | | **TD.96180 18% Protein diet** | | **TD.94266 50% protein diet** | |
| --- | --- | --- | --- | --- | --- | --- |
| **Formula:** | **g/Kg** | | **g/Kg** | | **g/Kg** | |
| Casein | 69 | | 207 | | 574.8 | |
| DL-methionine | 0.9 | | 2.7 | | 0 | |
| Sucrose | 571.8 | | 451.3 | | 131.5 | |
| Corn Starch | 200 | | 200 | | 200 | |
| Corn oil | 53.9 | | 52.6 | | 48.9 | |
| Cellulose | 57.82 | | 41.06 | | 2.65 | |
| Vitamin Mix, Teklad (40060) | 10 | | 10 | | 10 | |
| Ethoxyquin, antioxidant | 0.01 | | 0.01 | | 0.01 | |
| Mineral Mix, Ca-P Deficient (79055) | 13.37 | | 13.37 | | 13.37 | |
| Mineral Phosphate, dibasic | 21.6 | | 17.36 | | 6.07 | |
| Calcium Carbonate | 1.6 | | 4.6 | | 12.7 | |
|  |  | |  | |  | |
| **Nutrient information** | **% by weight** | **% kcal from** | **% by weight** | **% kcal from** | **% by weight** | **% kcal from** |
| Protein | 6.1 | 6.5 | 18.3 | 19.4 | 50 | 53.2 |
| Carbohydrate | 75.6 | 80.4 | 63.6 | 67.5 | 31.6 | 33.6 |
| Fat | 5.5 | 13.2 | 5.5 | 13.1 | 5.5 | 13.2 |
|  |  | |  | |  | |
| **Energy** | 3.8 Kcal/g | | 3.8 Kcal/g | | 3.8 Kcal/g | |

**Supplementary Table 1.** Tables of formula and nutritional information for 6% protein diet (TD.90016, Harlan, Teklad UK) , 18%protein diet (TD.96180, Harlan, Teklad UK) , 50% protein diet (TD.94266, Harlan, Teklad UK) (A) and cooked chicken breast (Sainsbury’s, UK) (B).

|  | |
| --- | --- |
| **Typical value** | **per 100 g** |
| Energy | 141 Kcal |
| Fat | 1.7 g |
| Carbohydrate | <0.5 g |
| Protein | 30.7 g |

1. Sainsbury’s’ British Chicken Fillets
